# Exploring risk transfer of human brucellosis in the context of livestock agriculture transition: A case study in Shaanxi, China

**DOI:** 10.3389/fpubh.2022.1009854

**Published:** 2023-01-27

**Authors:** Cuihong An, Li Shen, Minghao Sun, Yangxin Sun, Suoping Fan, Chenxi Zhao, Shoumin Nie, Boyan Luo, Ting Fu, Kun Liu, Zhongjun Shao, WenHui Chang

**Affiliations:** ^1^Department of Plague and Brucellosis, Shaanxi Center for Disease Control and Prevention, Xi'an, China; ^2^Department of Microbiology and Immunology, School of Medicine, Xi'an Jiaotong University, Xi'an, Shaanxi, China; ^3^School of Remote Sensing and Information Engineering, Wuhan University, Wuhan, China; ^4^Department of Epidemiology, Ministry of Education Key Lab of Hazard Assessment and Control in Special Operational Environment, School of Public Health, Air Force Medical University, Xi'an, China

**Keywords:** human brucellosis, agricultural intensification, foodborne infection, test-based methods, model-based methods, geographical detector

## Abstract

With the booming of worldwide agriculture intensification, brucellosis, one of the most neglected zoonotic diseases, has become an increasing challenge for global public health. Although the transmission patterns of human brucellosis (HB) have been studied in many regions, the dynamic transfer processes of risk and its driving factors remain poorly understood, especially in the context of agricultural intensification. This study attempted to explore the risk transfer of HB between the exact epidemic areas and the neighboring or distant low-risk areas to explain the impact of livestock agriculture intensification and foodborne infections on the transmission of HB in Shaanxi Province as a case study. We adopted multiple approaches, including test-based methods, model-based methods, and a geographical detector to detect the spatial-temporal dynamic changes of high-risk epidemic areas of HB at the county scale. We also quantitatively estimated how the related factors drove the risk transfer of the disease. Results confirmed the risk transfer pattern of HB with an expansion from north to south in Shaanxi Province and identified two primary transfer routes. In particular, in the traditional epidemic areas of the Shaanbei plateau, the farm agglomeration effect can significantly increase the risk of HB. Meanwhile, retail outlets for milk and dairy products were partially responsible for the foodborne infections of HB in the emerging epidemic areas of Xi'an. This study not only contributed helpful insights to support HB control and prevention in the rapid transition of livestock agriculture but also provided possible directions for further research on foodborne HB infections in urbanized areas.

## 1. Introduction

As a major global public health concern, emerging zoonotic diseases have been posing a serious threat to humans and animals, especially in developing countries (1). Human-dominated ecological variations, such as land use changes in agricultural intensification, were confirmed to be critical to affect the risk of zoonotic diseases and create expanding hazardous interfaces between people and livestock (2). Among those worldwide known zoonotic diseases, brucellosis, caused by the genus *Brucella*, is one of the most neglected bacterial zoonoses. Domestic animals are recognized as the main reservoir hosts of *Brucella*, including but not limited to sheep goats, and cattle. Humans can be infected through close contact with infected animals or eating contaminated animal products (3, 4). Recent evidence suggests that the risk of human brucellosis (HB) in developing countries has increased gradually due to agricultural intensification and rising animal production consumption (5, 6).

**Figure 1:**
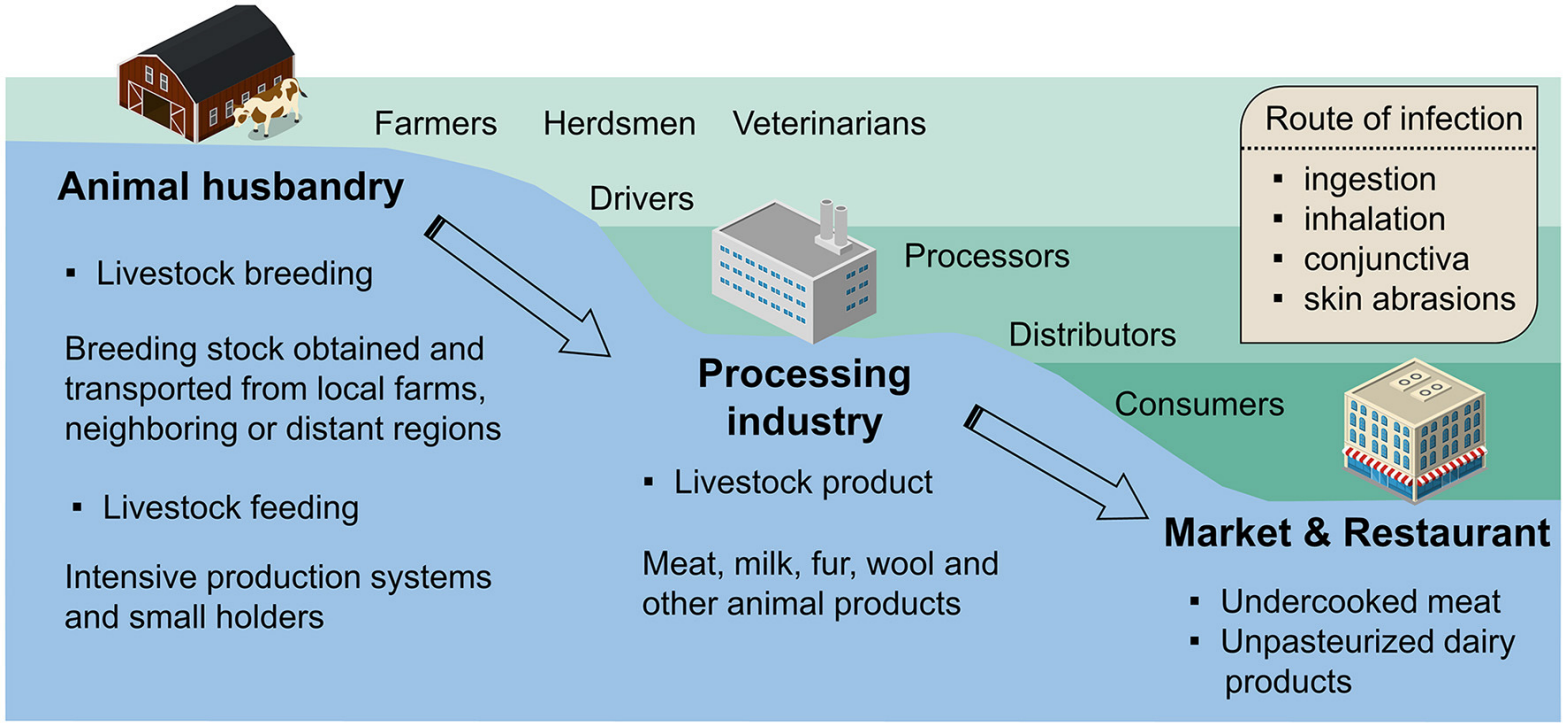
Conceptual framework for the transmission of human brucellosis. The transmission of pathogens is involved in breeding stock imports, livestock industry chain (black solid arrow), and poorly regulated trades (black dotted arrow).

Zoonotic risk transfer, the spatial and temporal dynamic changes, is probably affected by long-distance and frequent trades in intensive livestock agriculture and accessible animal-sourced foods (7). It can be seen from the conceptual framework in Figure 1 that the transmission of brucellosis risk exists in most parts of the livestock industry chain, and a variety of relevant occupations are at high risk of HB. First, in the breeding stage of animal husbandry, the import of breeding stock from other regions is an important source of brucellosis. Studies have shown that mounting unrestricted trades of livestock in rural areas can result in more HB-infected cases (8, 9). The brucellosis infections in the feeding stage are mainly related to the lack of protective measures when handling animals, which intensive farms perform better than smallholders. Next, in downstream industries, slaughterhouse processors and transport industry practitioners are likely to be exposed to infected livestock products. Finally, the endpoints of the brucellosis transmission chain are markets and consumers, where foodborne infection happens a lot and is caused by eating undercooked meat or unpasteurized dairy products. However, in complex supply networks, it is usually difficult to identify the source of contamination for foodborne infection, as brucellosis diffusion involves multiple levels of food producing and processing sites (10). This reduces the accuracy of backtracking trade data (10).

In recent years, there has been a rapid transition of livestock agriculture from the traditional free-range mode to the intensive producing mode in China (11), which is considered to possibly cause the re-emergence of brucellosis (12). Most studies have focused on extracting the spatial and temporal patterns when describing the epidemiological characteristics of brucellosis in China (13, 14) but have paid less attention to the dynamic transfer processes of HB risk. Furthermore, the impacts of intensive policies and livestock systems on brucellosis emergence have been explored in other countries such as Kazakhstan (15) and Nigeria (8), while in China the paucity of attention to the effects of livestock intensification on brucellosis emergence can be noted, particularly for some new types of livestock agricultural business (9).

Therefore, the aim of this study was to determine the transfer routes of HB risk around central urban agglomerations in Northwest China and to verify the impact of the new types of intensive farms and foodborne infection on HB in the context of livestock agriculture transition. We applied both statistical test-based and model-based methods to detect the risk transfer processes of HB and spatially and temporally examined the influence of driving factors using the geographical detector method. This study provided an in-depth exploration of risk transfer from high-risk areas to low-risk areas of HB and provided an understanding of zoonotic risk in the new intensive livestock industries. Due to the practical constraints of the available data, our study was implemented in Shaanxi Province, an epidemic area of HB in the northwest of China.

## 2. Materials and methods

### 2.1. Study area and materials

**Figure 2:**
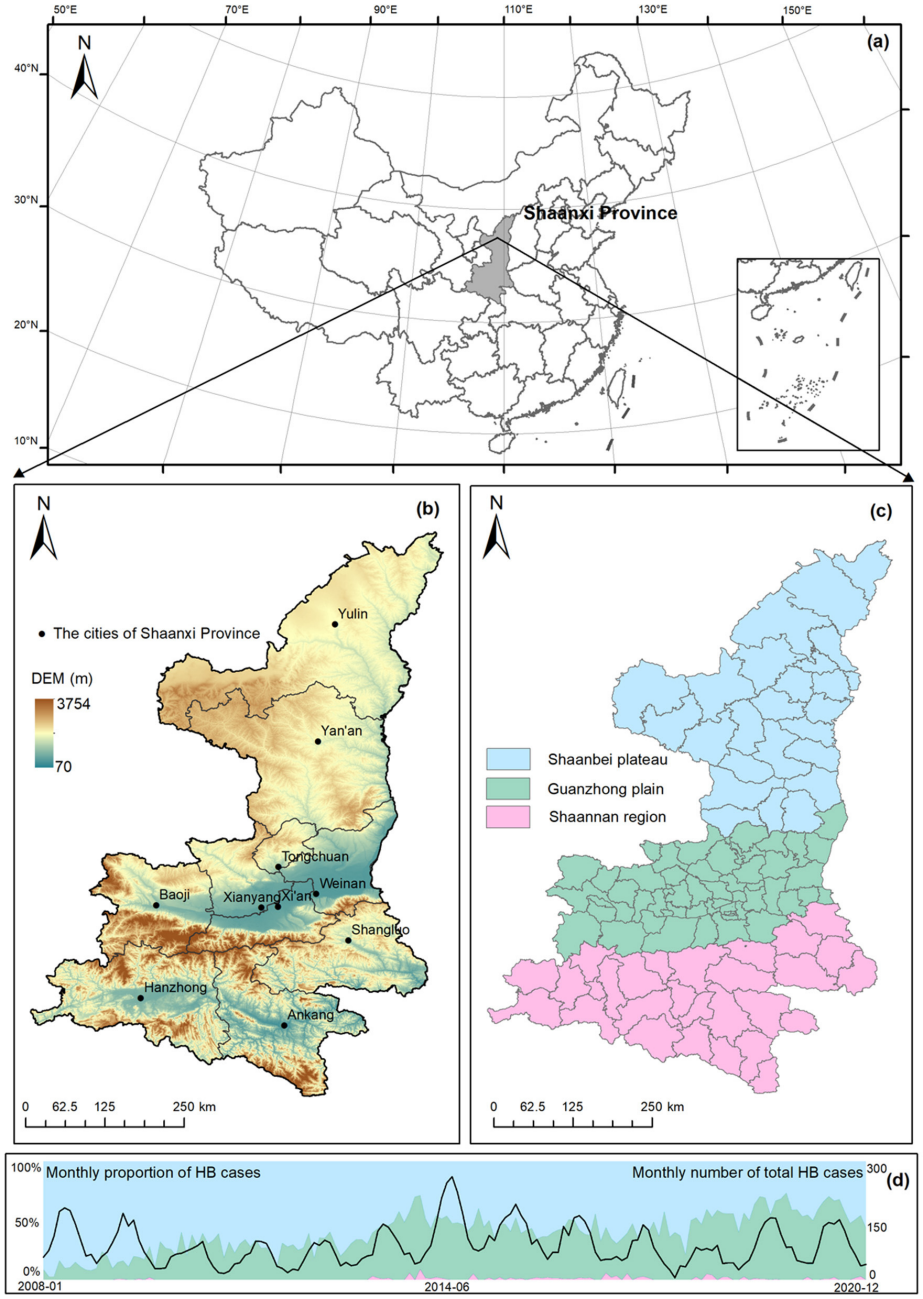
Study area and dynamic changes in HB cases. **(a)** Geographical location, **(b)** landforms and cities, **(c)** regional division, and **(d)** monthly number of HB cases in Shaanxi Province.

This study built an overall theoretical framework to explain the HB risk transfer processes, as shown in Figure 1. Shaanxi Province, located in the northwest of China (31°42′-39°35′N, 105°29′-115°15′E) (Figure 2a), consists of 10 prefecture-level cities and can be subdivided into 107 county-level divisions (Figure 2b). Shaanxi Province covers an area of more than 205,600 km^2^ with a permanent residential population of 39.53 million in 2022 (http://en.shaanxi.gov.cn/as/, 2022-04-15). Due to the distinct characteristics of climate and landforms, Shaanxi Province is divided into three continuous geographical regions, including the Shaanbei plateau (northern), the Guanzhong plain (central), and the Shannan region (southern) (Figure 2c). As one of the most severely affected regions by highly prevalent brucellosis, Shaanxi Province reported a total number of 13,502 confirmed HB cases from 2005 to 2020 (16). The total number of HB cases in the Shaanxi Province peaked in 2014, and the proportion of HB cases in the Shaanbei plateau demonstrated a wavelike declining trend over time (Figure 2d). In contrast, in recent years, there has been a continual growth of HB cases in the Guanzhong plain, as noted by the previous studies (16, 17), which pointed out that the Guanzhong plain has become an emerging high-risk area of the HB epidemic. Therefore, it is imperative to investigate how the risk of HB transfers between the three geographical regions in Shaanxi.

The data on diagnosed HB cases in Shaanxi from 2008 to 2020 was provided by the Center for Disease Control and Prevention of Shaanxi Province through the National Notifiable Infectious Diseases Reporting Information System. The livestock statistical data obtained from the Statistical Yearbook of Shaanxi Province Bureau of Statistics (http://tjj.shaanxi.gov.cn/, 2022-04-15) was processed to calculate Spearman's Rank Correlation Coefficients with the incidence rate of HB. The variables with statistically significant correlations were selected as the input data for those model-based methods. According to the discussion in relevant studies by Hu et al. (18), we selected large-scale family farms, demonstration farmers' specialized cooperatives, and industrial leading enterprises above the designated size as the three fundamental types of intensive livestock business in the study area, which was acquired from the online database provided by the Department of Agriculture and Rural Affairs of Shaanxi Province (http://nynct.shaanxi.gov.cn/agriresources/?source=management, 2022-04-15). Supplementary Figure S1a offered an overview of the spatial distribution of those new intensive livestock businesses. The number of livestock in the 107 counties presented as a base map consists mainly of cattle, goats, and sheep. It is apparent that the Shaanbei upland plateau is a typical crisscross zone of livestock keeping and farming, where animal husbandry has rapidly developed, while in the economically developed Guanzhong plain located the most industrial leading enterprises that meet the huge consumption of milk and meat products. As the Shannan region is characterized by mountainous landforms and hills, the number of farmers' specialized cooperatives in the Shannan region is much lower compared to that in the Shaanbei upland and Guanzhong plain. In consideration of the high population density and urbanization level in Xi'an city, the capital of Shaanxi Province, we selected it as the target area to examine the impacts of foodborne HB infection. Information on retail outlets and mobile vendors, two types of businesses involved in the sale of livestock products sale possibly contaminated with *Brucella*, was sourced from the Food Safety Public Enquiry System of Shaanxi Province (http://gkgs.sxfda.gov.cn/, 2022-04-15). As shown in Supplementary Figure S1b, retail outlets were almost located in central urban areas, while mobile vendors were concentrated in the suburbs of Xi'an city. With a few exceptions, the cases of brucellosis foodborne infection occurred near retail outlets.

### 2.2. Methods

**Figure 3:**
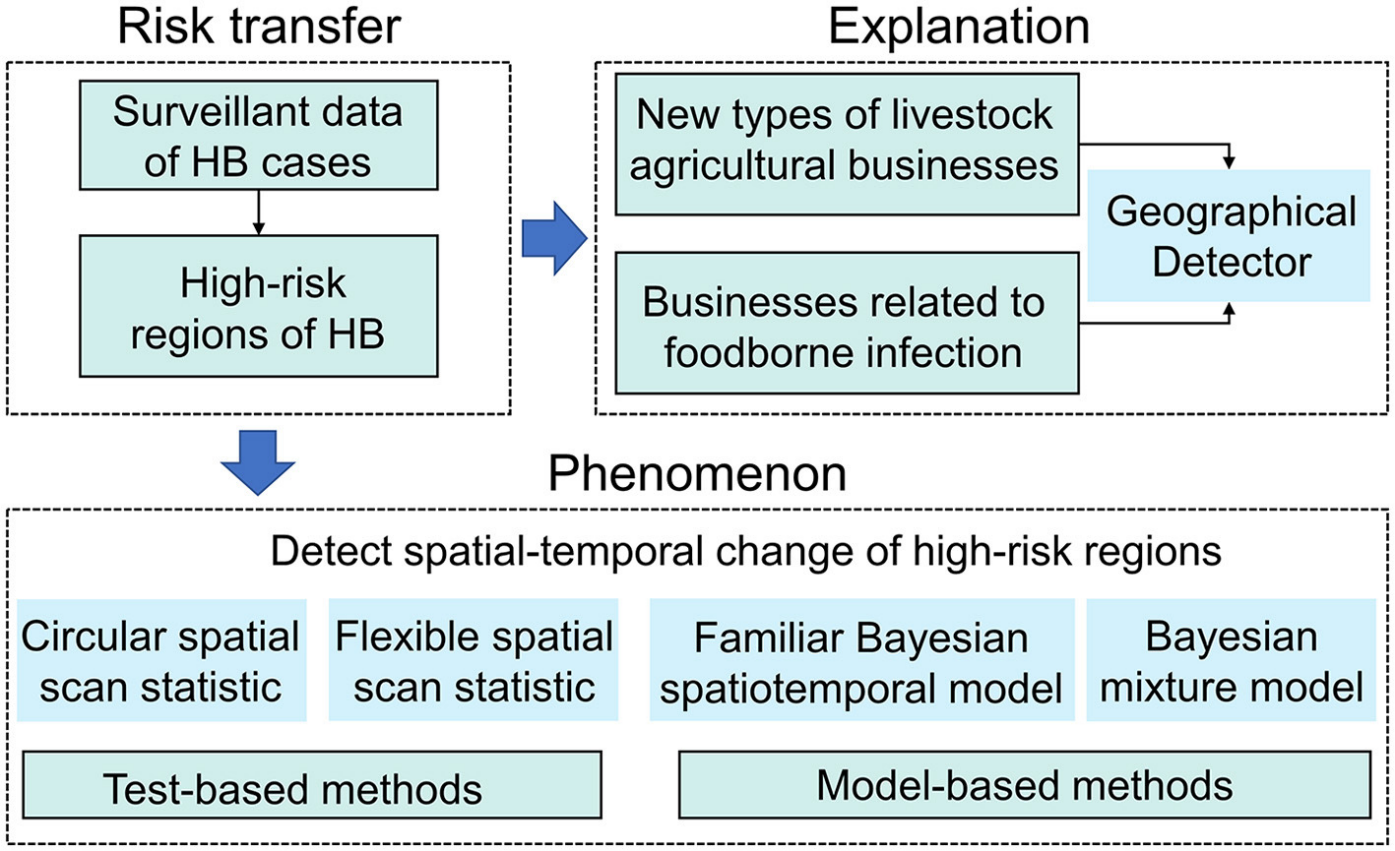
Research methods used to investigate the phenomenon and explanation of HB risk transfer. Test-based methods could provide spatiotemporal distribution of clusters in high-risk areas of HB and model-based approach can estimate posterior relative risk changes of HB. Geographical detector could quantify the explanatory power of impact factors on HB spatial heterogeneity.

This study examined the risk transfer of human brucellosis from the perspective of both phenomenon and reasons. Local risk in low-risk areas may increase due to livestock trade with endemic areas, which is reflected in the increasing incidence of HB. Therefore, we first detected areas with a high incidence of HB and determined the changes in their spatial locations. To detect changes more comprehensively in high-risk areas, we applied both methods based on scan statistics (the circular spatial scan statistic and the flexible spatial scan statistic) and model-based methods (the familiar Bayesian spatiotemporal model and the Bayesian mixture model). Next, we identified the driving factors of the risk transfer. After examining the characteristics of brucellosis transmission in Shaanxi, we selected two factors that were strongly correlated with HB (businesses related to livestock agricultural intensification and foodborne infection) and quantified their impact based on the geographical detector (GeoDetector). A flowchart of the process was presented in Figure 3.

#### 2.2.1. Test-based methods

Since most test-based methods for cluster detection identify regions with relatively identical characteristics based on scan statistic, spatial circular and flexible scan statistics have been widely used to investigate spatial or space-time disease clusters (19). The key point of the spatial circular scan statistic is the moving scan window and likelihood ratio test statistic. The space-time cylindrical scan window Z has a circular geographic base and a height corresponding to time. With a dynamic center, the circular base traverses each region, and the height covers any possible time interval in the study period. The radius ranges from 0 to the predetermined maximum range. Let *n*_*Z*_ and *N*_*Z*_ denote the number of observed cases and populations at risk in zone Z, respectively. *n* and *N* denote the total number of observed cases and populations at risk, respectively. The log-likelihood ratio (LLR) test statistic λ is calculated for each cylinder based on these numbers (20), which is given by the following equation:


(1) $$\begin{array}{l} \text{λ}=\frac{L\left(Z\right)}{L_{0}} \end{array}$$

(2) $$\begin{array}{l} L(z)=\{\begin{array}{c} {\left(\frac{n_{Z}}{N_{Z}}\right)}^{n_{Z}}{\left(1-\frac{n_{Z}}{N_{Z}}\right)}^{N_{Z}-n_{Z}}{\left(\frac{n-n_{Z}}{N-N_{Z}}\right)}^{n-n_{Z}}{\left(1-\frac{n-n_{Z}}{N-N_{Z}}\right)}^{\left[\left(N-N_{Z}\right)-\left(n-n_{Z}\right)\right]},\;if\;\frac{n_{Z}}{N_{Z}}>\frac{n-n_{Z}}{N-N_{Z}}\\ {\left(\frac{n}{N}\right)}^{n}{\left(\frac{N-n}{N}\right)}^{\left(N-n\right)},\;if\;\frac{n_{Z}}{N_{Z}}\leq \frac{n-n_{Z}}{N-N_{Z}} \end{array} \end{array}$$

Where *L*_0_ denotes the null hypothesis that depends on the total number of cases (21). Monte Carlo simulation is used for the significance test, and the highest test statistic refers to the most likely cluster with the highest relative risk (*RR*). Other clusters with high likelihood values are classified as secondary likely clusters.

Compared with the spatial circular scan statistic, the flexible scan statistic performs more efficiently in capturing clusters in flexible shapes but is merely appropriate for panel data as the input. Since this method was first proposed by Tango And Takahashi (22), the log-likelihood ratio test statistic has been improved to avoid the limited number of near neighbors (23). The restricted likelihood ratio statistic is defined as


(3) $$\begin{array}{l} \begin{array}{l} {\text{λ}}_{T}\left(Z\right)={\left(\frac{n_{Z}}{\xi_{Z}}\right)}^{n_{Z}}{\left(\frac{n-n_{Z}}{n-\xi_{Z}}\right)}^{n-n_{Z}}I\left(\frac{n_{Z}}{\xi_{Z}}>\frac{n-n_{Z}}{n-\xi_{Z}}\right)\;\\ \;\prod_{i\in Z}I\left(p_{i}<\alpha_{1}\right) \end{array} \end{array}$$

Where ξ_*Z*_ denotes the number of expected cases in *Z*, and *p*_*i*_ is the one-tailed *p*-value of the test for the null hypothesis (23). Like the original scan statistic, the *p*-value is obtained through Monte Carlo simulation, and the most satisfying clusters can be consequently determined.

To further reveal the specific routes and process of HB risk transfer, we examined the peak periods in the monthly time series of HB cases. In addition, to examine the differences between the two scan statistic methods in detecting potential HB clusters, we applied the SaTScan v10.0 (https://www.satscan.org/) and FleXScan v3.1.2 (https://sites.google.com/site/flexscansoftware/) software with the total diagnosed HB cases and population for the 107 counties in the Shaanxi Province as model inputs. We detected clusters, respectively, in the aforementioned three geographical regions through the SaTScan software, and the flexible scan statistic of the FleXScan software was used to detect clusters in key regions that changed obviously annually. The maximum radius of the scan window in SaTScan was set to 30% of the total populations at risk, and the maximum number of neighbors in FleXScan was set to the default value of 15.

#### 2.2.2. Model-based methods

Since the Markov Chain Monte Carlo (MCMC) method was effectively developed and applied, the Bayesian spatiotemporal model has been a relevant research direction in spatial epidemiology. Researchers have built some widely used Bayesian models, such as Besag-York-Mollié (BYM) model, the conditional autoregressive (CAR) model, and the Bayesian hierarchical model (24). Particularly in disease mapping, the CAR model is regarded as a commonly used framework for detecting spatiotemporal changes in relative risk (25). Compared to the scan statistic test-based methods, model-based methods are more appropriate for extracting information on spatiotemporal interaction and analyzing multiple covariates such as risk factors. In addition, a variety of prior information can support optimizing those models and improving the outputs. In this article, we selected two typical Bayesian models that were proposed in recent years, including the familiar Bayesian spatiotemporal model (FBM) and the Bayesian mixture model for HB risk detection.

Referring to the hypothesis proposed by Li et al. (26), we assumed that the observed HB cases *y*_*it*_ of area *i* at time *t* follow a Poisson distribution, which is modeled as


(4) $$\begin{array}{l} y_{it}\sim Poisson\left(n_{i}\mu_{it}\right) \end{array}$$

*n*_*i*_ denotes the number of populations at risk in area *i*, and the relative risk of HB μ_*it*_ is given by


(5) $$\begin{array}{l} \log\left(\mu_{it}\right)=\alpha +s_{i}+b_{0}t^{{}^{*}}+v_{t}+\beta x_{i}+b_{1i}t^{{}^{*}}+\varepsilon_{it} \end{array}$$

Where α is the logarithm of overall risk, and t^*^ is the difference between *t* and the median of our study period. Term *s*_*i*_ measures the spatial distribution of disease risk. ($b_{0}t^{*}+v_{t}$) and $b_{1i}t^{*}$ can be, respectively, interpreted as the common time trend and local time trend in area *i*. *x*_*i*_ represents various covariates and β is the corresponding coefficient. ε_*it*_ denotes the random error term.

A two-stage classification method was also proposed by Li et al. (26). In the first stage, area *i* is defined as a hot spot or a cold spot based on the posterior probability of spatial risk term *s*_*i*_. Then, the posterior probability of the rate of local risk change *b*_1*i*_ is used to identify the local time trend of increasing and decreasing variation. Other areas of posterior probabilities in the usual interval have common trends consistent with the overall variation.

The Bayesian mixture models are characterized by a similar fundamental specification that *y*_*it*_ follows a Poisson distribution, while *n*_*i*_ is replaced by the number of expected HB cases *E*_*it*_. Besides, μ_*it*_ is modeled as the combination of secondary models with a common risk $\mu_{it}^{\left(C\right)}$ and an area-specific risk $\mu_{it}^{\left(AS\right)}$. The mixing parameter *z*_*it*_ satisfies a hierarchical Bernoulli prior distribution (27). This mixture model is defined as follows:


(6) $$\begin{array}{l} \log\left(\mu_{it}\right)=z_{it}\mu_{it}^{\left(C\right)}+\left(1-z_{it}\right)\mu_{it}^{\left(AS\right)} \end{array}$$

(7) $$\begin{array}{l} \mu_{it}^{\left(C\right)}=\alpha_{0}+\eta_{i}+\gamma_{t} \end{array}$$

(8) $$\begin{array}{l} \mu_{it}^{\left(AS\right)}=\nu_{i}+\kappa_{it} \end{array}$$

In the common model, η_*i*_ and γ_*t*_ describe the overall spatial distribution and temporal changes, respectively, and α_0_ is the intercept term. In the local model, ν_*i*_ is the intercept of local relative risk, and κ_*it*_ measures the time trend in area *i* at time point *t*. When the value of *z*_*it*_ is 1 or 0, the common model or the local model will be accordingly selected as the estimate of the relative risk. To detect the unusual observations, the posterior frequency that *z*_*it*_ takes 1, *f*_*it*_ = *P*(*z*_*it*_ = 1|*data*) has been calculated. At the standard statistical significance level of 0.05, the *f*_*it*_ less than 0.95 indicates that the trend pattern of area *i* at time point *t* follows a local trend. Other areas at other time points would be identified as usual observations (27, 28).

As for the prior distribution of model parameters in this study, some key spatial parameters or hierarchical parameters, such as *s*_*i*_ and η_*i*_, follow a CAR prior or an intrinsic conditional autoregressive prior (ICAR). Other parameters with less knowledge follow a weakly informative prior, such as intercept terms. Most prior distributions of other parameters remain the default settings in the original models (26, 27).

We implemented both models in OpenBUGS v3.2.3 (29) for detecting areas with unusual temporal patterns. Two MCMC chains with different initial values were set for each of these two models. A total of 100,000 iterations were run, and 25,000 iterations were burned in at the beginning of the chains. By sampling every 25 iterations, autocorrelation was reduced. We diagnosed the convergence through trace plots and the Gelman–Rubin statistic (30), which approximated 1 for all parameters. The iteration results were finally processed to calculate the posterior probabilities in R 4.0.4.

#### 2.2.3. GeoDetector

GeoDetector was first proposed by Wang et al. (31) based on the theory of spatial stratified heterogeneity (SSH). The simple and convenient properties of GeoDetector contribute to its wide application in environmental and health problems. One of the main tasks of GeoDetector is to quantitatively measure the driving forces behind geographic phenomena. The *q*-statistic is used to test the coupling relationship between two categorical variables *X* and *Y*, which is calculated as follows:


(9) $$\begin{array}{l} q=1-\frac{\sum_{h=1}^{L}N_{h}\sigma_{h}^{2}}{N\sigma^{2}} \end{array}$$

Where *N*_*h*_ and $\sigma_{h}^{2}$ denote the number of sampling units and the variance of *Y* in the study area *h*, respectively. *L* is the number of strata, which can be determined by *X*. The *q*-statistic measures the linear and non-linear associations between the explanatory variable *X* and the response variable *Y*. *Q*-statistic = 0 indicates no coupling associations existing between variables *X* and *Y*; *q*-statistic = 1 indicates that *X* can explain 100% of the stratified heterogeneity in *Y* (31). Furthermore, GeoDetector is more feasible in a relatively small set of sample data compared to other classical statistic methods (32). It can provide favorable conditions for analyzing the impacts of foodborne infection on human brucellosis risk.

To explore the impact of livestock agricultural intensification on HB risk transfer, *Y* is interpreted as the HB cases by non-foodborne infection in statistical units of 5 km grids in the three geographical regions. We assumed that the distance between the geometrically central point of each grid, and the relevant explanatory variables included points for the three types of livestock agriculture businesses. Based on the above results, we found Xi'an to be a potential city for foodborne transmission. We used GeoDetector again to further explore the effect of foodborne transmission on HB risk transfer. *Y* is the number of HB cases by foodborne infection in statistical units of 1 km grids in Xi'an city, and the point of goat milk and dairy products sale location is the explanatory variable. Because the explanatory variables were numerical, we used the Natural Breaks tool in the ArcGIS 10.2 software to convert them to categorical variables. We implemented this method through the GeoDetector software based on Excel 2007 (33). Furthermore, after calculating the distances between HB case points and the five types of points (three kinds of agricultural points and two kinds of foodborne points), we plotted the distribution of their probability densities in 2017 and 2018 using the Python library seaborn 0.11.1.

### 2.3. Ethical statement

In China, the collection of data from brucellosis cases is part of routine public health surveillance, and such data collection is exempt from institutional review board assessment. Ethical approval for this study was not required in accordance with local legislation and national guidelines.

## 3. Results

### 3.1. Results of test-based methods

Clusters detected by SaTScan are basically categorized into two types as follows: one is characterized by gathering the central points of different counties in circle shapes and the other is for isolated points in a certain single county (see Supplementary Figure S2 for their spatial and temporal distributions). Cluster 1 (LLR = 1736.72, RR = 4.93, *P*-value < 0.001) included six counties in the Shaanbei plateau and covered a period from 2008 to 2013, indicating a significant high-risk area of HB in the Shaanbei plateau in the early study period. The secondary detected area in the Shaanbei plateau was an isolated point, Cluster 2 (LLR = 80.85, RR = 2.88, *P*-value < 0.001), possibly due to the explosion of HB cases in 2014 throughout the whole Shaanxi Province. In the Guanzhong plain and Shannan region, five clusters were found after 2012. Those clusters can clearly exhibit the increasing trend of HB cases in central-southern Shaanxi Province in recent years. The strong adjacency relationship and chronological order suggested that the HB risk possibly transferred from the previous high-risk areas to the emerging epidemic areas in two regions. One included Cluster 3 (LLR = 2071.78, RR = 8.57, *P*-value < 0.001) and Cluster 6 (LLR = 193.56, RR = 23.48, *P*-value < 0.001), while the other one comprised Cluster 4 (LLR = 197.55, RR = 3.71, *P*-value < 0.001) and Cluster 5 (LLR = 449.94, RR = 5.96, *P*-value < 0.001).

**Figure 4:**
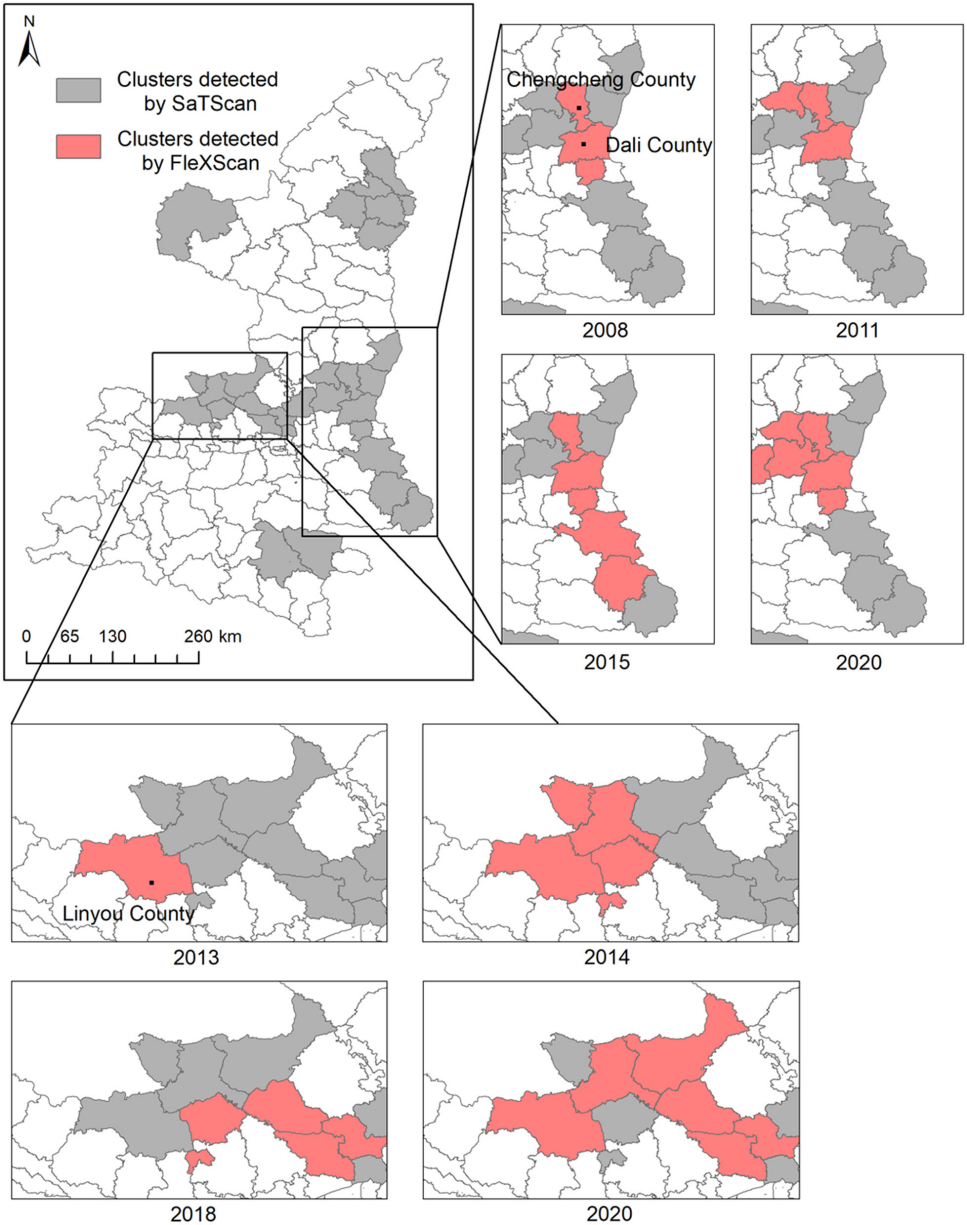
Spatial clusters detected by FleXScan in two key regions in Shaanxi Province.

To further identify the transfer route of HB risk, more specific results in the two aforementioned regions have been provided by flexible scan statistical analysis. The clusters detected by FleXScan (Figure 4) indicated that the HB risk transfer did exist between these counties, which have been detected as clusters by SaTScan. In those two regions framed by rectangles, the most likely clusters (*P*-value < 0.001) for some key years were presented in the form of panel data. The long and narrow strip-shaped expansion from north to south covered the whole study period in the right fringe of Shaanxi Province, especially showing the largest in 2015 when there was an obvious uptrend of HB cases in Guanzhong plain and Shannan region. Results also revealed that counties (Chengcheng County and Dali County) in the north of this strip region are likely to cover the local HB risk sources. In contrast, the detected cluster in the left region of Guanzhong Plain showed a more complicated expansion with an irregular shape in later years. Since 2013, HB risk has appeared relatively higher in Linyou County than in surrounding areas. This cluster of high HB risk continuously expanded in the next 7 years, gradually covering the economic and political center of Shaanxi Province from the fringe to the inner area. Clusters detected by SaTScan can provide the general spatial-temporal pattern of HB risk transfer, while clusters detected by FleXScan can help interpret the possibly dynamic routes of HB risk transfer.

**Figure 5:**
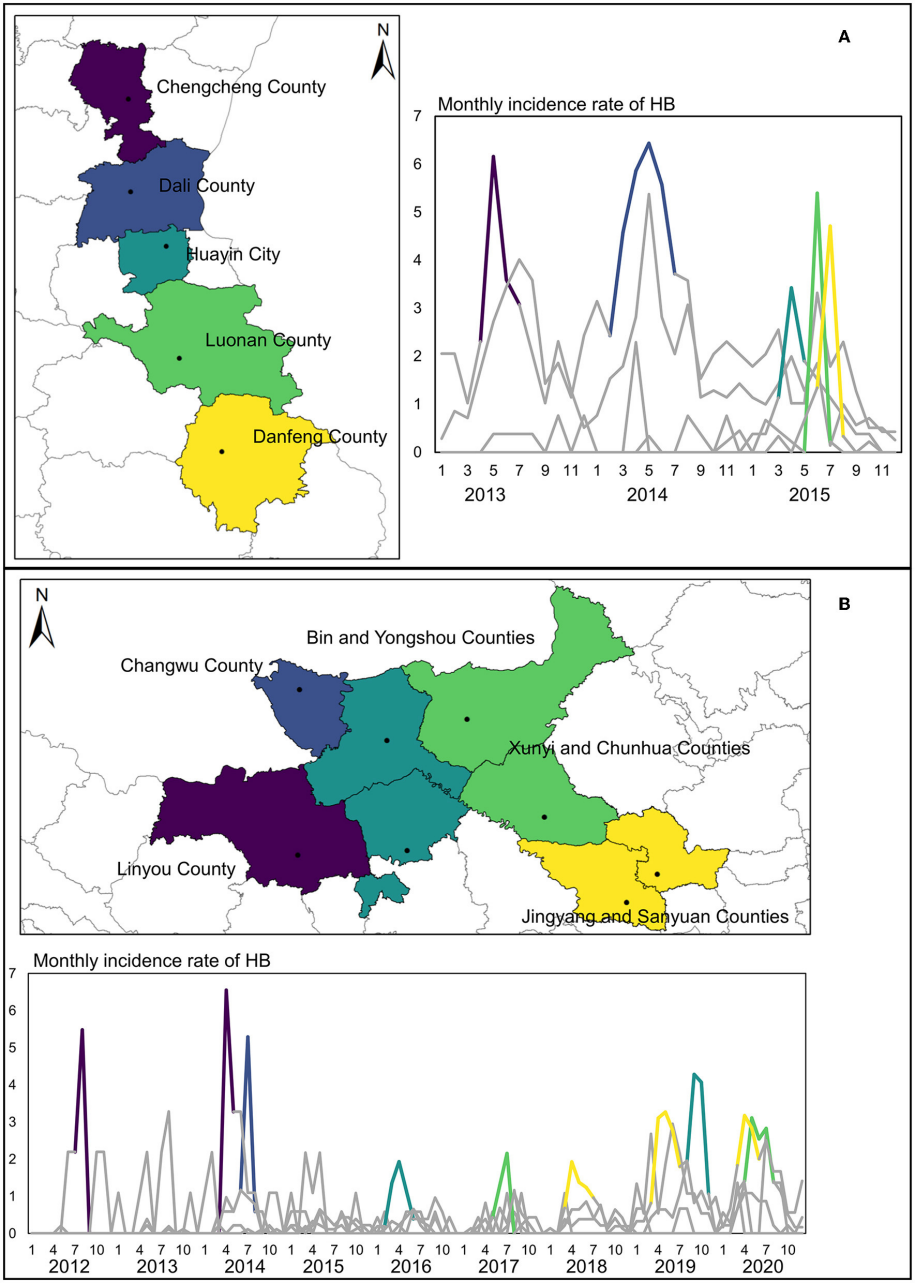
Monthly time series of HB cases and peak periods in two key regions. **(A)** Peaks of the time series of HB cases in the Weinan-Shangluo region. **(B)** Peaks of the time series of HB cases in the left region of the Guanzhong Plain.

Although a comparison between these two methods based on different scan statistics can provide a general interpretation of the HB risk transfer from high-risk areas to the surrounding areas in the two regions, we further inspected the peak periods in the monthly time series of HB cases to reveal the specific routes and processes of HB risk transfer in those two regions. Figure 5A illustrates the counties in the HB transfer route in different graduated colors, which corresponded to the specific peak period in the monthly time series for each individual county. It was found that the peaks successively moving southward occurred in three consecutive years 2013, 2014, and 2015. Specifically, the number of HB cases in Chengcheng County peaked first in 2013 and then in Dali County in 2014. Despite the peak times of the other three counties (Huayin, Luonan, and Danfeng) in the same year, 2015, a difference in this particular month did exist. In this strip-shaped cluster, the HB risk transfer is much more likely to occur between Weinan city (Chengcheng, Dali, and Huayin) in Guanzhong plain and Shangluo city (Luonan and Danfeng) in the Shannan region.

For another irregular cluster shown in Figure 5B, the risk of HB was found to transfer from two possible sources of Linyou County and Changwu County, which differed from the Weinan-Shangluo region. For other counties in this cluster, the peak period of HB cases apparently occurred in chronological order. What is noteworthy is that those counties are located in two cities that both belong to the western fringe area of Shaanxi Province, indicating a higher transfer possibility from outside of Shaanxi Province rather than directly from the Shaanbei plateau in the north of Shaanxi Province. Consequently, the yielded findings suggested that there existed two possible transfer routes of HB risk in two primary geographical clusters. However, the causal relationships among those counties in the HB risk transfer routes are difficult to quantify in this study, due to the lack of detailed infection information in the HB cases. Moreover, the impacts of adjacent areas outside of Shaanxi Province should also be considered in analyzing the HB risk transfer routes. Such restrictions and problems are elaborated extensively in the discussion section. In summary, scan statistic test-based methods are useful tools in efficiently detecting the possible transfer routes of HB risk to support decision-making in disease control.

### 3.2. Results of model-based methods

**Figure 6:**
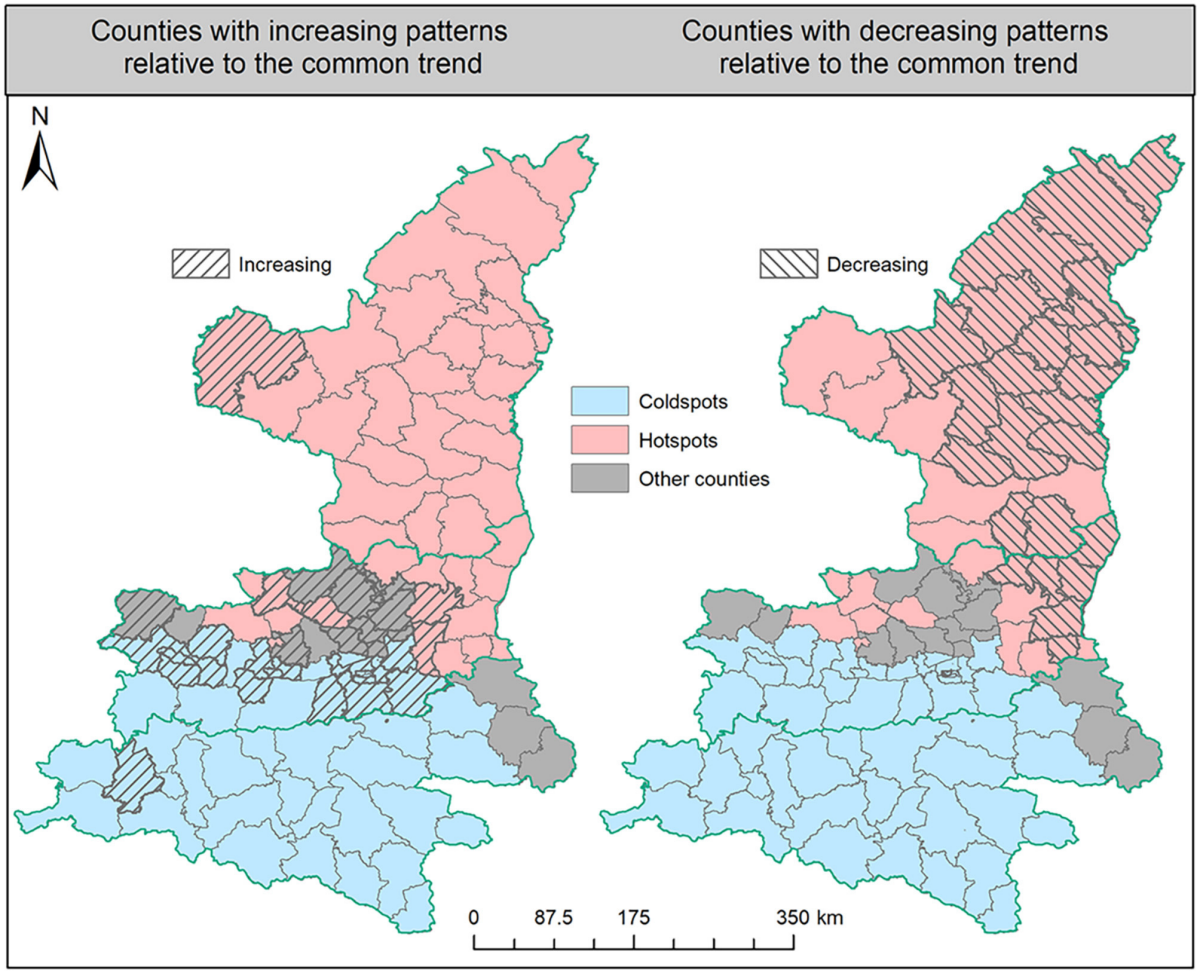
Hot spots and cold spots with different temporal patterns.

The results of the FBM analysis could demonstrate the dynamic characteristics of HB risk from the two primary perspectives of spatial aggregation and temporal variation. Concerning the spatial patterns, Figure 6 shows all counties in Shaanxi Province that were first identified as hot spots, cold spots, or others. Counties in the Shaanbei plateau and some in the Guanzhong plain showed a higher spatial risk of HB than the average level in the whole province. In addition, in the Shannan region, there were only three counties in Shangluo city not identified as cold spots, and those three counties partly contributed to the detected strip-shaped transfer route of HB risk. It is more interesting to note that other counties with neither hot nor cold spots partly formed the dividing line between the hot spot counties in northern Shaanxi Province and the cold spot counties in the south. This further confirmed the expansion process of HB risk from the northern Shaanxi Province toward the south. With respect to the temporal variation, we compared the dynamic local trends of each individual county to the common trend of the entire Shaanxi Province. From the left map in Figure 6, it is clearly found that counties with increasing patterns were almost located in the Guanzhong plain and Shannan region. By contrast, from the right map in Figure 6, most counties in the Shaanbei plateau exhibited decreasing patterns compared to the common trend, although the absolute number of HB cases in some of those counties rose during the study periods. However, the FBM method has limited ability to effectively capture the specific local outliers from the common trend in the time series for each county, and how to accurately measure the common trend of HB cases in Shaanxi Province is another challenging task for the FBM.

**Figure 7:**
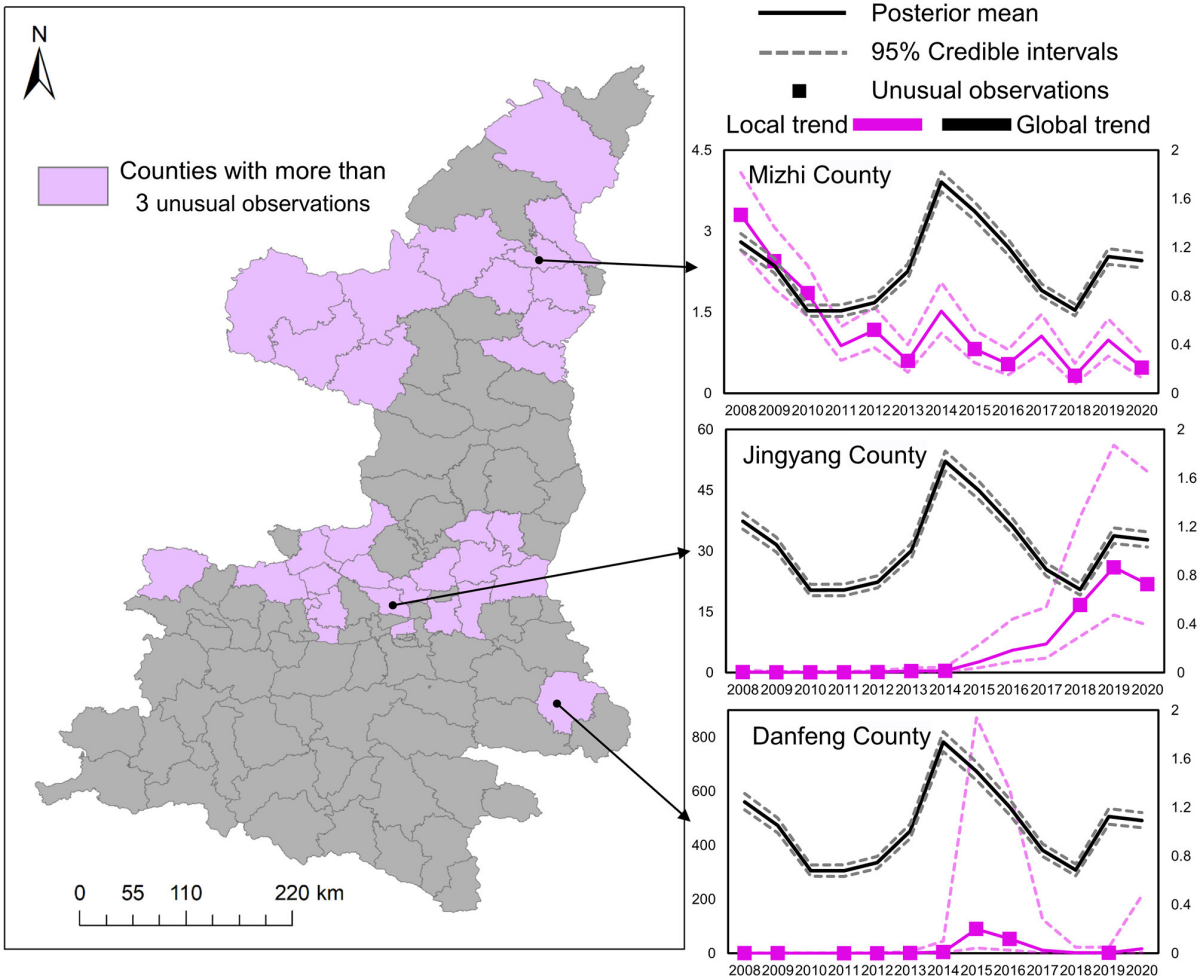
The difference between local trends and the global common trend.

To overcome the aforementioned limitations, we further applied the mixture Bayesian model to more specifically estimate the differences between the local trends and the global common trend of HB cases. Results in Figure 7 demonstrate the specific locations of counties with more than three unusual observations detected by the mixture Bayesian model in more detailed temporal variation. Different from the clusters detected by the SaTScan method, the unusual observations found by the mixture Bayesian model included both increasing and decreasing trends of HB cases. Owing to the widespread decreasing trends of HB cases, more observations of counties were identified as unusual in the Shaanbei plateau. Moreover, we selected three counties (Mizhi, Jingyang, and Danfeng) as examples to show how to analyze the typical patterns of local trends based on posterior statistics. Based on the curves of the global trend in Figure 7, it can be found that there was an obvious peak of HB risk in 2014 in Shaanxi Province. As one typical county with a decreasing pattern of HB cases, Mizhi County almost had a consistent trend with the common patterns in HB incidence. In the curves for Jingyang County, it is interesting to observe the rapid increase of HB cases after the peak year of 2014, and Jingyang County was also the last node of the previously detected transfer route. In Danfeng County, the incidence rate of HB peaked in 2015 and remained steady at a low level in other years. In brief, scan statistics test-based methods and model-based methods can provide useful insights into the risk transfer routes of HB cases between high-risk areas and low-risk areas.

### 3.3. Impact of livestock agricultural intensification and foodborne infection

**Figure 8:**
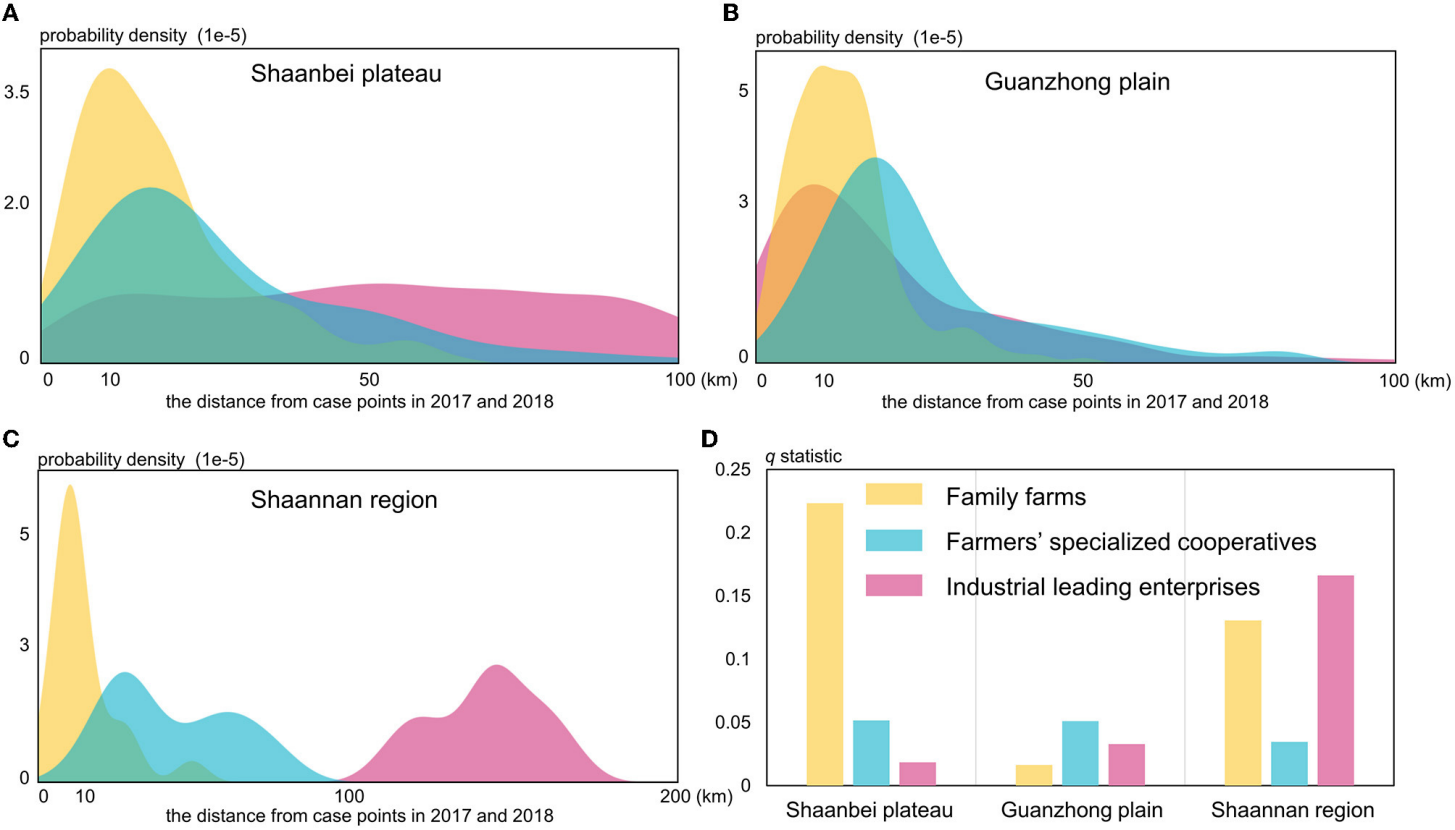
The probability density distributions in three regions of Shaanxi Province **(A–C)** and *q*-statistic values **(D)** for the livestock agricultural variables.

According to the *q*-statistic values (*P*-value < 0.001) yielded by GeoDetector, the impacts of three livestock agricultural variables (family farms, farmers' specialized cooperatives, and industrial leading enterprises) on the spatial distribution of HB cases were quantitatively measured. It is found that the Shaanbei plateau family farms (*q*-statistic = 0.223) had the strongest influence on the spatial distribution of HB cases (Figure 8D). The extracted probability density distribution in the Shaanbei plateau indicated that most HB cases were located within ~10 km of family farms, which was similar to the characterized distribution of HB case points in Guanzhong plain and Shannan region (Figures 8A, B). Similarly, in the Shannan region, family farms were also found to have a significant impact on the distribution of HB cases, and industrially leading enterprises (*q*-statistic = 0.166) were regarded as the most important factors (Figure 8D). Since the distances between industrially leading enterprises and HB case points were obviously >100 km, these enterprises could possibly cause negative effects on the distribution of HB cases in the Shannan region (Figure 8C). It is interesting that the *q*-statistics of family farms appeared much smaller in the Guanzhong plain compared to that in both the Shaanbei plateau and Shannan region (Figure 8D). In addition, farmers' specialized cooperatives and industrial leading enterprises in the Guanzhong plain could merely explain <10% of the total stratified heterogeneity of HB cases, which confirmed that the primary driving factors of the spatial distribution of HB cases in Guanzhong plain could be hardly associated with livestock agricultural intensification. To further examine this interesting phenomenon, we conducted a Geodector analysis to detect the foodborne infection specifically in Xi'an city, the primary metropolitan in Guanzhong plain.

**Figure 9:**
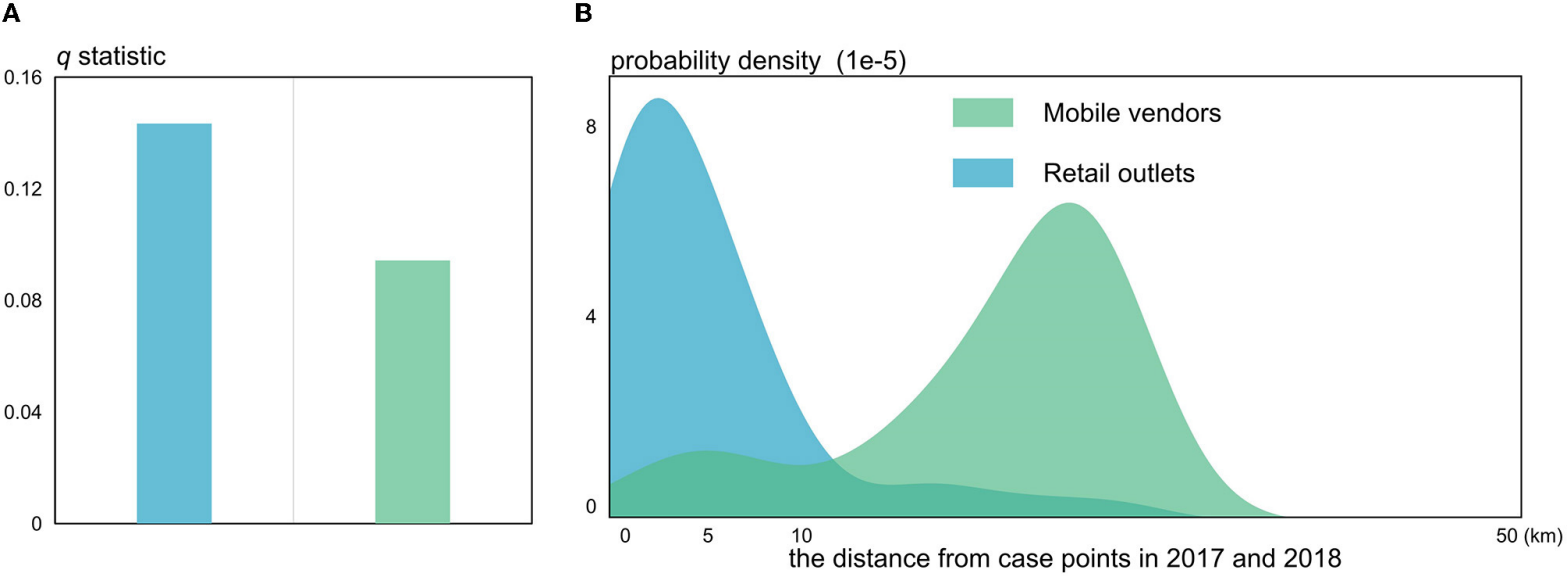
The *q*-statistic **(A)** and probability density distributions **(B)** for the foodborne variables.

The *q*-statistic values for retail outlets (*q*-statistic = 0.143, *P*-value < 0.001) and mobile vendors (*q*-statistic = 0.094, *P*-value < 0.001) shown in Figure 9A were purposed to measure the impact caused by the foodborne infection in Xi'an city. With the distance from HB case points continuously increasing, the probability density of retail outlets peaked at <5 km while that of mobile vendors peaked at much more than 10 km from the HB case points (Figure 9B). In other words, it is apparently indicated that retail outlets can be considered more important in determining the foodborne infection of HB compared to mobile vendors. This is possibly due to the fact that most HB cases by foodborne infection were closely linked with or influenced by a booming retail industry in the populated urban centers. However, due to the strong flexibility of mobile vendors selling raw goat milk and dairy products, there is no sufficient evidence proving that mobile vendors are unable to increase the risk of HB cases through foodborne infection. Those results, combined with the previous ones, can provide some preliminary and useful insights into the driving forces and causes of the risk transfer of HB in Shaanxi Province.

## 4. Discussion

This study spatiotemporally explored the impacts of new livestock agricultural policies on HB by quantitatively examining the risk transfer of HB in Shaanxi Province. One of the important results revealed that the risk transfer of brucellosis appeared noticeably after 2013, which can be closely related to the prevention and control strategies of brucellosis at the grass-roots level that cannot adapt to the rapid development of agricultural development. Especially in Shaanxi Province, agricultural industrialization has been greatly promoted since 2012, and family farms were later implemented in 2013 (34), engendering the dramatic intensification of livestock in subsequent years. These findings suggested that more attention should be paid to the conflicts and trade-offs between agricultural intensification and zoonotic disease prevention. Nevertheless, the dynamic changes in brucellosis risk are of great uncertainty, and the lag effect was impacted by a wide range of influential factors such as anthropogenic damaging intervention in the natural ecosystem, the development of transregional trade networks, as well as the intensification of livestock agriculture (17, 35). In addition, some transactions that evade regulation and insufficient quarantine of animal production in rapidly developed animal husbandry industrialization are also partially responsible for HB emergence and transfer particularly caused by foodborne infection (36). In spite of some environmental factors such as rainfall, temperature, and vegetation that can also impact the transmission mechanism of brucellosis (37, 38), it should come as no surprise that anthropogenic activities play a key role in determining the emergence and transfer of HB. Because different study areas have diverse driving factors of HB transfer concerning their own specific livestock agricultural policies and geographical circumstances, it must be recognized that quantitative analysis conducted to examine brucellosis risk transmission in one place may not be appropriate for applying directly to another area. In other words, it is difficult to systematically investigate the brucellosis risk transfer in a comprehensive way to consider all existing influential aspects. However, this study was performed as a preliminary and tentative study that can be deepened by integrating more influential factors of HB risk transfer. More endeavor is needed to understand different geographical contexts to improve the accuracy of the result as well as efficiency.

Besides, both scan statistic test-based (circular and flexible spatial scan statistic) and model-based (FBM and mixture model) methods were utilized to explore HB risk transfer as well as to measure the influence of significant driving factors. Our analysis found the scan statistic test-based methods have great potential for effectively and easily detecting the HB high-risk areas, while their shortages in spatiotemporally capturing variation can be well overcome by the model-based methods. The advantages of using multiple approaches lie in improving accuracy and reducing subjective judgments for HB risk detection. Admittedly, it still cannot avoid some limitations associated with the use of those multiple methods due to the difficulty in precisely extracting the quantitative cause-effect relationships between different factors and HB risk transfer. As we know, significant statistical linkages do not necessarily imply a causal relationship (39). Therefore, it is imperative to obey certain strict criteria in determining a causal relationship. As suggested by Hill (40), concerns from nine perspectives can serve as the fundamental checklist of causal effect criteria, which point out that results based on randomized experiments are more reliable and valid than observational studies (41). However, it is difficult for us to conduct such randomized experiments at present owing to restrictions in research funding, data accessibility, and brucellosis complexity. Another chance to accurately estimate causal effect is the approach developed by Google works based on the Bayesian structural time series (BSTS) model with a designed invention in a time series (42). In this study, we attempted to use this method to extract the spatial transfer routes of HB risks, and potential routes in two key clusters have been detected, but how and why these risks were transferred remained unknown for further investigation.

In summary, the endeavor in this study is considered a necessary attempt toward an accurate examination of HB risk transfer. The results strengthen the ideas that livestock agriculture intensification has played a vital role in the increase of HB risk, and the combination of test-based methods and model-based methods can be extended to other emerging zoonoses. Furthermore, our findings can provide some constructive suggestions for brucellosis prevention and control. First, the animal quarantine department needs to improve the supervision of cross-regional trade and set a reasonable isolation period for imported livestock. Second, large-scale breeding plants should establish their own internal sources of breeding stock, and smallholders should also reduce the introduction of livestock in an epidemic area. Finally, new occupations in animal husbandry must strictly adhere to the personal protection system, and consumers should avoid purchasing livestock products from informal channels.

As an interdisciplinary research, this study not only addressed some research gaps identified in existing literature but was also limited by several unavoidable restrictions, such as the absence of more recorded HB cases in longer time series at different geospatial scales. For example, some sub-transfer routes were impossible to detect at the county scale in our study. Therefore, multi-scale adaptive methods were a vital and promising direction in our further research. Besides, another inevitable uncertainty was the HB risk transferred from the adjacent regions outside Shaanxi Province, mainly including the two neighboring provinces of Shanxi and Gansu (43), where the epidemic situations of HB were considered less optimistic and several cities (Yuncheng City and Tianshui City) were also identified as high-risk areas. In general, cross-regional cooperative research and interdisciplinary approaches were regarded as practical solutions to solve such problems, and this would be another consideration in our further study.

## 5. Conclusion

The main purpose of this study was to determine the risk transfer routes of human brucellosis and the effects of livestock intensification in Shaanxi Province, China. Two key transfer routes have been identified, and our findings support the hypothesis that the intensification of livestock agriculture as well as foodborne infection can increase the risk of human brucellosis in emerging epidemic areas under the current epidemic situation. Our findings also provided new evidence for the expansion of HB into and around regional central cities during the transitional development of animal husbandry. Further studies are needed to establish a more comprehensive framework of methodologies for HB risk detection and driving factors analysis by considering multi-scale and neighboring effects.

## Data availability statement

The raw data supporting the conclusions of this article will be made available by the authors, without undue reservation.

## Ethics statement

Ethical review and approval was not required for the study on human participants in accordance with the local legislation and institutional requirements. Written informed consent for participation was not required for this study in accordance with the national legislation and the institutional requirements. Written informed consent was not obtained from the individual(s) for the publication of any potentially identifiable images or data included in this article.

## Author contributions

Conceptualization: ZS, KL, and WC. Investigation, resources, and data curation: CA, YS, SF, SN, and BL. Methodology, visualization, and formal analysis: LS, MS, CZ, and TF. Writing—original draft: CA, LS, and MS. Writing—review and editing: KL, TF, and ZS. All authors contributed to the article and approved the submitted version.
