# Supplementary material for: Exploring risk transfer of human brucellosis in the context of livestock agriculture transition: A case study in Shaanxi, China
Source: Front Public Health. 2023 Jan 27;10:1009854. doi: 10.3389/fpubh.2022.1009854 (PMC9911661; doi:10.3389/fpubh.2022.1009854)
Supplement: Supplementary file 1 [file Data_Sheet_1.docx]

***Supplementary materials*：**

**Exploring risk transfer of human brucellosis in the context of livestock agriculture transition: A case study in Shaanxi, China**

Cuihong An ^1,2†^, Li Shen ^3†^, Minghao Sun ^3†^, Yangxin Sun ^1^, Suoping Fan ^1^, Chenxi Zhao ^4^, Lipeng Yang ^3^, Shoumin Nie ^1^, Boyan Luo ^1^, Ting Fu ^4^, Kun Liu ^4*^, Zhongjun Shao ^4*^, WenHui Chang ^1*^

# Supplementary Figures


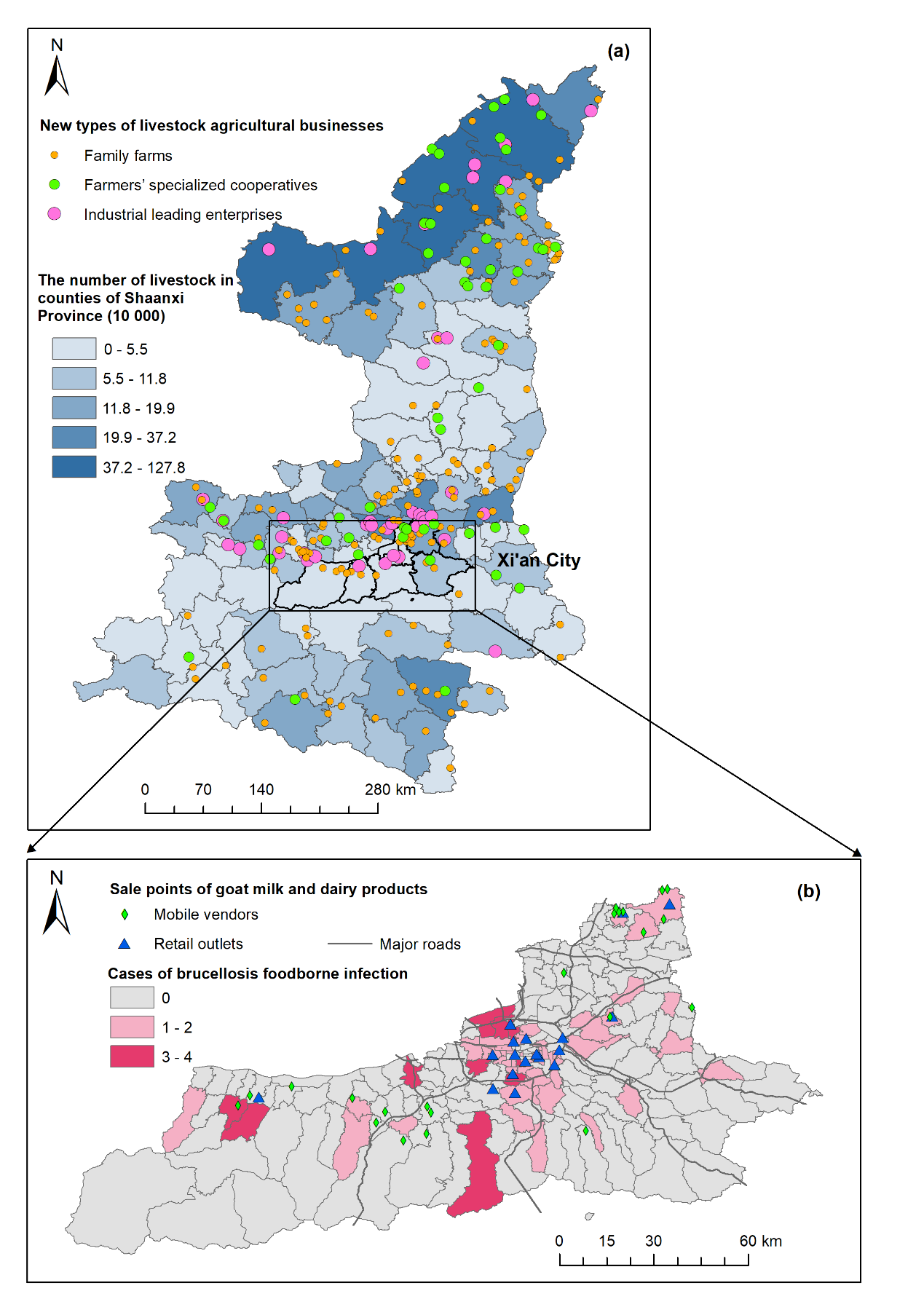


**Fig.S1 Influencing factors of livestock intensification and foodborne infection in Shaanxi province.** (a) Spatial distribution of new intensive livestock businesses. (b) Spatial distribution of two types of businesses in livestock products sale.


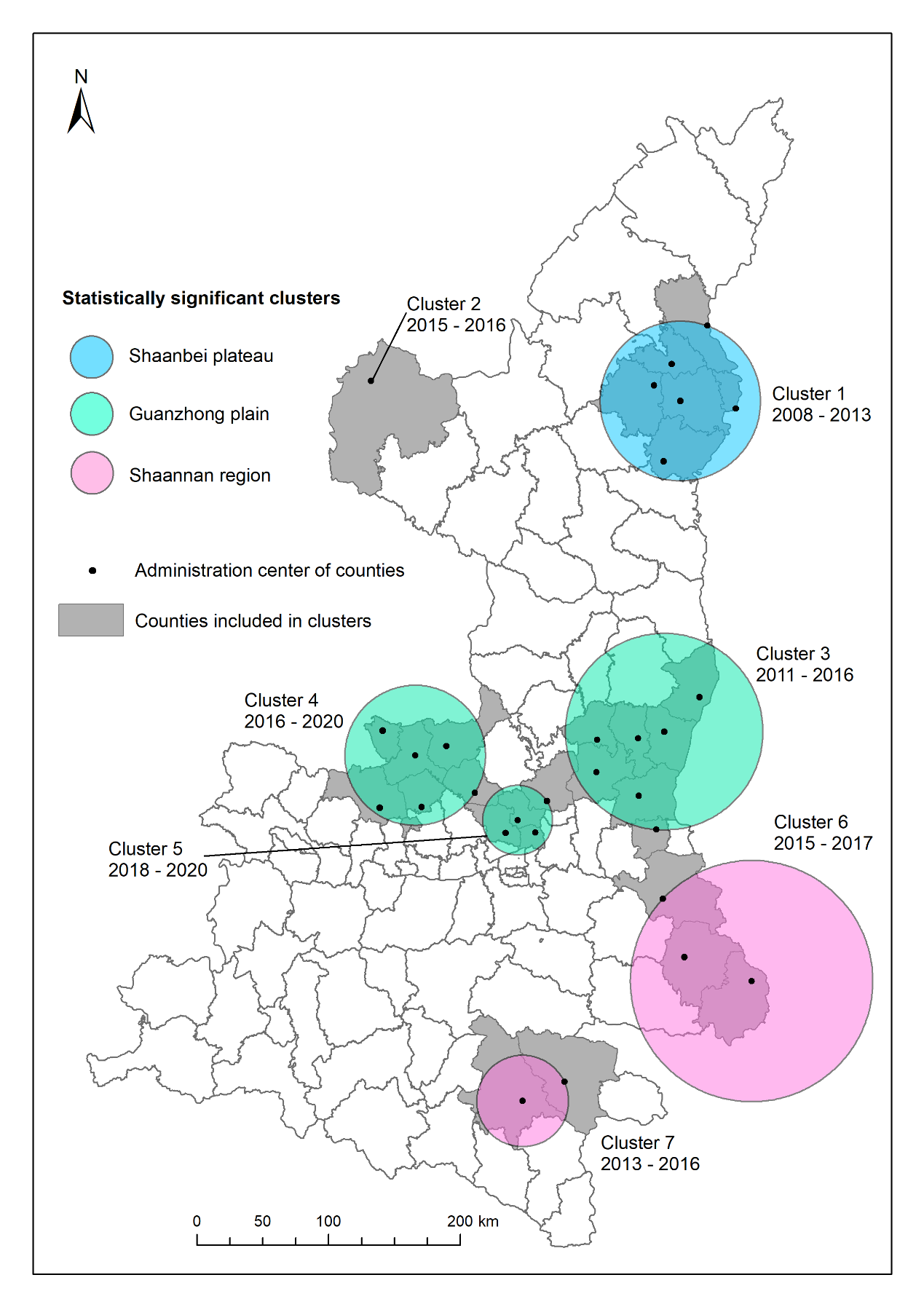


**Fig.S2** **Statistically significant clusters detected by SaTScan in three geographical regions in Shaanxi, China.**
